# Multiple chronic conditions and associated health care expenses in US adults with cancer: a 2010–2015 Medical Expenditure Panel Survey study

**DOI:** 10.1186/s12913-019-4827-1

**Published:** 2019-12-19

**Authors:** Mary Lynn Davis-Ajami, Zhiqiang K. Lu, Jun Wu

**Affiliations:** 1 Department of Science of Nursing Care, Indiana University School of Nursing, 1033 East Third Street, Bloomington, IN 47045 USA; 2 Department of Clinical Pharmacy and Outcomes Sciences, University of South Carolina College of Pharmacy, 715 Sumter St., Columbia, SC 29208 USA; 3 Department of Pharmaceutical and Administrative Sciences, Presbyterian College School of Pharmacy, 307 N. Broad St., Clinton, SC 29325 USA

**Keywords:** Cancer, Multiple chronic conditions, Health expenses, Health utilization

## Abstract

**Background:**

Cancer increases the risk of developing one or more chronic conditions, yet little research describes the associations between health care costs, utilization patterns, and chronic conditions in adults with cancer. The objective of this study was to examine the treated prevalence of chronic conditions and the association between chronic conditions and health care expenses in US adults with cancer.

**Methods:**

This retrospective observational study used US Medical Expenditure Panel Survey (MEPS) Household Component (2010–2015) data sampling adults diagnosed with cancer and one or more of 18 select chronic conditions. The measures used were treated prevalence of chronic conditions, and total and chronic condition-specific health expenses (per-person, per-year). Generalized linear models assessed chronic condition-specific expenses in adults with cancer vs. without cancer and the association of chronic conditions on total health expenses in adults with cancer, respectively, by controlling for demographic and health characteristics. Accounting for the complex survey design in MEPS, all data analyses and statistical procedures applied longitudinal weights for national estimates.

**Results:**

Among 3657 eligible adults with cancer, 83.9% (*n* = 3040; representing 16 million US individuals per-year) had at least one chronic condition, and 29.7% reported four or more conditions. Among those with cancer, hypertension (59.7%), hyperlipidemia (53.6%), arthritis (25.6%), diabetes (22.2%), and coronary artery disease (18.2%) were the five most prevalent chronic conditions. Chronic conditions accounted for 30% of total health expenses. Total health expenses were $6388 higher for those with chronic conditions vs. those without (*p* < 0.001). Health expenses associated with chronic conditions increased by 34% in adults with cancer vs. those without cancer after adjustment.

**Conclusions:**

In US adults with cancer, the treated prevalence of common chronic conditions was high and health expenses associated with chronic conditions were higher than those without cancer. A holistic treatment plan is needed to improve cost outcomes.

## Background

Living with cancer or surviving cancer increases the risk of multiple chronic conditions (MCCs) such as hypertension, diabetes, and depression due to undergoing treatment, demographics, and other behavioral factors [1–4]. Comorbid chronic conditions occur in about seven out of 10 cancer patients [5]. The presence of any chronic condition, particularly multiple chronic conditions, complicates cancer treatment and increases health care costs and productivity loss [6, 7]. Mental health conditions, heart disease, stroke, and diabetes significantly contribute to higher annual health care expenses in cancer patients [8–10]. The presence of a greater number of comorbidities is associated with higher health care expenses [11, 12], creating a substantial economic burden for cancer patients.

The majority of prior research assessed the economic impact of mental health conditions in cancer patients [10, 13, 14]. The knowledge of how other common chronic conditions, such as cardiovascular diseases, chronic obstructive pulmonary disease (COPD), and asthma, affect economic outcomes is limited [9, 10]. Most studies focused on total health care costs, yet few measured the costs of specific common chronic conditions in cancer patients, or compared these costs between those with cancer vs. those without cancer. Knowing the total health care costs incurred from chronic conditions and how the presence of one or more chronic conditions is associated with health care utilization patterns is important in determining a model of care that addresses collateral comorbid chronic care issues. This information may help health system decision-makers determine the skills that specialty and primary care health care providers need to address the complex needs of cancer patients with MCC. Further, understanding the prevalence of chronic conditions and associated outcomes in the adult cancer population compared to the general population gives insight into how to optimize treatment, address patient satisfaction, and minimize the economic burden for patients and payers.

The objectives of this study were to estimate the treated prevalence of commonly occurring MCC, and to assess the association between chronic conditions and health care utilization and costs in US adults with cancer.

## Methods

### Data source

This is a retrospective study using the US Medical Expenditure Panel Survey (MEPS) Household Component data (2010–2015), administered by the Agency for Healthcare Research and Quality (AHRQ). MEPS is a subsample of households that participated in the previous year’s National Health Interview Survey, forming a nationally representative sample of the non-institutionalized US population. The survey uses an overlapping panel design, including five rounds of interviews. Each panel covers a two-year timeframe. During the interview, MEPS collects detailed information for each member of the household, including demographic characteristics, health conditions, health status, medical service utilization, medication use, payments, and insurance coverage. Interviewers record medical conditions and health care utilization as reported by survey participants. This information is then confirmed by the survey respondent’s medical provider, and coded by professional coders into appropriate diagnostic codes [15]. This study used the most recent publicly available longitudinal data files: panels 15 (2010–2011), 16 (2011–2012), 17 (2012–2013), 18 (2013–2014), and 19 (2014–2015), as well as the medical condition and event files to identify eligible respondents. The longitudinal files only included participants who completed all five interview rounds. This full MEPS participation status is represented in the data by a longitudinal weight greater than zero. MEPS provides information on medical conditions associated with a medical event. This association precludes unreported or undiagnosed medical conditions; therefore, MEPS data reports treated prevalence and not disease prevalence [16].

### Study sample

The study sample was derived by combing the MEPS longitudinal data from panels 15–19 and included respondents aged ≥18 years. Individuals with missing values in the survey were excluded. This group of eligible adults were then divided into two groups: those with any cancer diagnosis, and those with no cancer diagnosis. The cancer group were further divided into chronic and non-chronic condition groups for subgroup analysis. All cancer conditions were identified using Clinical Classification Software (CCS) codes (011–043) in the medical condition files. CCS for ICD-9-CM is a research tool developed by the Healthcare Cost and Utilization Project (HCUP), sponsored by AHRQ, that aggregates ICD-9-CM condition codes into clinically meaningful categories by grouping similar conditions [17]. To classify chronic conditions, we used the classification scheme developed by the US Department of Health and Human Services (HHS) Office of the Assistant Secretary of Health (OASH) and used by five HHS data systems, including MEPS. The HHS standard classification identifies 20 chronic conditions with their corresponding diagnosis codes [18]. In this study, CCS codes or ICD-9-CM codes were used to identify 18 chronic conditions. Cancer, the studied condition, and autism, which had extremely low prevalence, were excluded from the list of chronic conditions in this study.

### Outcome measures

The primary outcomes were per-person, per-year health expenses (medical and prescription drugs) for each two-year panel. Medical expenses were identified from the following medical event files: physician office visits, inpatient and outpatient visits, emergency department (ED) visits, and home care visits. The prescription drug expenses were identified from the prescribed medicines files. All health expenses summed the amounts paid by insurance and survey respondents and were inflated to the year 2015 using the personal consumption expenditure health indexes [19].

This study measured two types of health expenses: all-cause total health care expenses for any medical condition, and expenses related to chronic conditions. The medical condition and event files were linked and the CCS codes were used to identify disease-specific medical expenses for each chronic condition. The medical condition and prescribed medicines files were linked using CCS codes to identify prescribed medications associated with specific chronic conditions.

The secondary outcomes included the treated prevalence of individual chronic conditions, number of chronic conditions (0, 1, 2, 3, ≥4), and health care utilization patterns, measured by numbers of office, outpatient, hospitalization, and ED visits, prescriptions filled, and days provided by home health. We used ICD-9-CM diagnosis codes to identify individual chronic conditions based on medical events. Thus, the prevalence of the chronic condition was treated prevalence.

### Statistical analysis

Chi-square tests compared the prevalence of chronic conditions in adults with cancer vs. those without cancer. Among those with chronic condition(s), independent t-tests compared chronic condition-related health care utilization and expenses (total, medical, and prescription drug) in adults with cancer vs. without cancer. For the subgroup of adults with cancer, chi-square tests compared sociodemographic characteristics in those with chronic condition(s) vs. those without chronic condition(s); independent t-tests compared total health care utilization and expenses in those with chronic conditions vs. those without chronic conditions. Generalized linear model (with log link and gamma distribution) assessed the influence of cancer (yes vs. no) on chronic condition-related health expenses in all eligible adults with chronic condition(s) and the influence of chronic condition(s) (yes vs. no) on total health expenses in the subgroup of adults with cancer respectively by controlling for sociodemographic and health characteristics. Statistical significance was set a priori at *p* < 0.05. Accounting for the complex survey design in MEPS, all data analyses and statistical procedures applied longitudinal weights to produce national estimates and were performed by using SAS version 9.4.

## Results

**Fig. 1:**
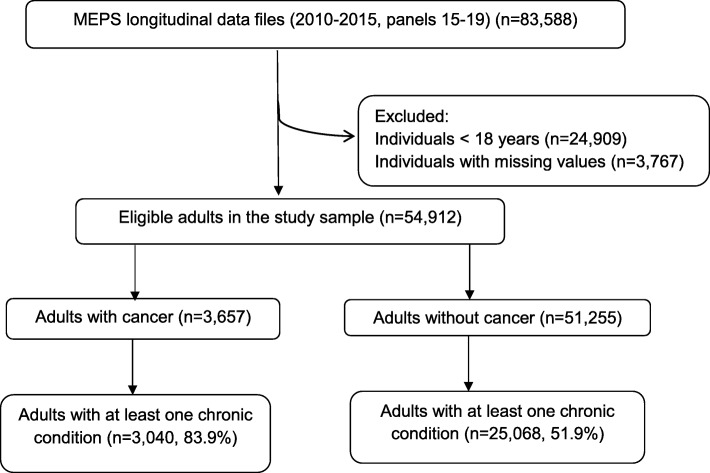
Study sample of eligible adults

The study sample included 3657 eligible adults with cancer, representing 19.1 million individuals in the US per-year during years 2010 to 2015. Among the eligible adults with cancer, 83.9% (*n* = 3040; representing 16 million individuals in the US) had at least one chronic condition (Fig. 1). Table 1 compared the characteristics of the adults with cancer and chronic conditions vs. adults with cancer but without chronic conditions. Overall, compared to the group without chronic conditions, adults with chronic conditions showed greater proportions of those reporting age older than 65 years (59.2% vs. 23.3%, *p* < 0.001), obesity (31.9% vs. 17.7%, *p* < 0.001), education lower than college (67.8% vs. 57%, *p* < 0.001), poor to low income (30.5% vs. 21.3%, *p* < 0.001), public insurance coverage (29.9% vs. 14.4%, *p* < 0.001), and poor to fair health status (23.6% vs. 11.2%, *p* < 0.001).

**Table 1:** Characteristics by chronic conditions in adults with cancer (*n* = 3657)

Variable	Chronic condition (*n* = 3040)	No chronic condition (*n* = 617)	*p*
	n (weighted %)	n (weighted %)	
Age category			< 0.001
18–45	300 (7.8)	219 (30.5)	
46–64	1024 (33.0)	263 (46.2)	
65+	1716 (59.2)	135 (23.3)	
Race			0.006
White	1872 (74.5)	359 (69.3)	
Black	368 (5.4)	56 (4.6)	
Other	800 (20.1)	202 (26.2)	
Sex (female)	1645 (57.8)	374 (56.5)	0.178
Body mass index			< 0.001
Underweight	45 (1.3)	19 (2.6)	
Normal	901 (31.6)	265 (45.0)	
Overweight	1061 (35.1)	210 (34.8)	
Obese	1033 (31.9)	123 (17.7)	
Education			< 0.001
High School	1720 (57.0)	319 (49.9)	
College	814 (32.2)	224 (43.0)	
No degree/other	506 (10.8)	74 (7.1)	
Family size			< 0.001
≤ 2	2355 (81.6)	354 (65.6)	
> 2	685 (18.4)	263 (34.4)	
Geographical region			0.259
Northeast	515 (17.9)	93 (15.3)	
Midwest	649 (23.0)	131 (22.8)	
South	1191 (38.6)	206 (36.8)	
West	685 (20.5)	187 (25.1)	
Married	1681 (59.6)	394 (65.7)	0.034
Income category^a^			< 0.001
Poor	670 (16.3)	97 (11.1)	
Low income	475 (14.2)	82 (10.3)	
Middle income	806 (25.7)	170 (24.7)	
High income	1089 (43.9)	268 (53.9)	
Insurance coverage			< 0.001
Private	1817 (66.9)	444 (77.3)	
Public	1084 (29.9)	115 (14.4)	
Uninsured	139 (3.2)	58 (8.3)	
Perceived health status			< 0.001
Excellent/Very good	1224 (45.3)	399 (70.7)	
Good	943 (31.2)	134 (18.1)	
Fair/poor	873 (23.6)	84 (11.2)	

^a^Poor defined as income < 100% of federal poverty line (FPL); low income defined as 100–199% of FPL; middle income defined as 200–399% of FPL; high income defined as ≥400% of FPL

**Fig. 2:**
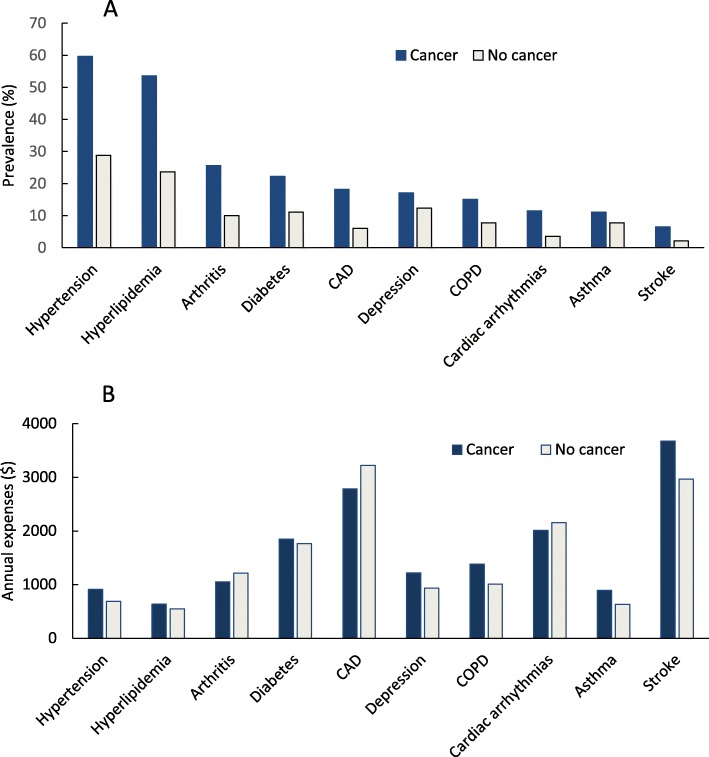
Prevalence (**a**) and annual health expenses (per-person, **b**) associated with common chronic conditions in adults by cancer CAD: coronary artery disease; COPD: Chronic Obstructive Pulmonary Disease. COPD: chronic obstructive pulmonary disease.

**Fig. 3:**
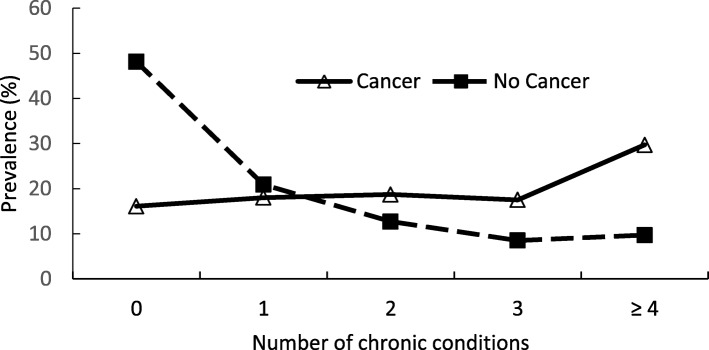
Distribution of chronic conditions number by cancer (*n* = 54,921)

Figure 2a indicated that the prevalence of common chronic conditions in adults with cancer was higher than in those without cancer. Hypertension (59.7%), hyperlipidemia (53.6%), arthritis (25.6%), diabetes (22.2%), and coronary artery disease (18.2%) were the five most prevalent chronic conditions in US adults with cancer. Additional file 1 lists all chronic conditions, ranked by treated prevalence, in the study sample. Among the top ten prevalent treated chronic conditions, stroke ($2964), coronary artery disease ($2787), and cardiac arrhythmias ($2011) were the costliest in adults with cancer (Fig. 2b). Additional file 2 shows annual expenses associated with all individual chronic conditions in cancer and no cancer groups. The proportions of adults with zero, one, two, and three chronic conditions in the cancer group were similar, ranging from 16.1 to 18.7%. However, 29.7% of the adults with cancer reported four or more chronic conditions vs. 9.7% of those without cancer (Fig. 3).

Table 2 compared chronic condition-related health care utilization and expenses by cancer. Overall higher health care utilization except ED visits were observed in the cancer group. The medical and total expenses associated with chronic conditions in the cancer group were $1061 ($2990 vs. $1929, *p* < 0.001) and $1399 ($4113 vs. $2714, p < 0.001) higher than in no cancer group. The health expenses associated with chronic conditions accounted for 30.3% of total all-cause health expenses. Prescription drug use, hospitalization, and office visits accounted for 27.3, 26.8, and 24.4%, respectively, of the chronic condition-related expenses in the cancer group.

**Table 2:** Chronic condition-related health service utilization patterns and expenses (per-person per-year) by cancer (*n* = 28,108)

	Cancer (n = 3040)	No cancer (*n* = 25,068)	*p*
Chronic condition-related health service utilization, mean (SE)			
Number of office visits	4.8 (0.2)	3.2 (0.1)	< 0.001
Number of outpatient visits	0.4 (0.1)	0.2 (0.0)	< 0.001
Number of prescriptions	6.7 (0.2)	4.6 (0.1)	< 0.001
Home health days per month	0.4 (0.1)	0.3 (0.0)	0.002
ED visits per 1000 patient months	7 (0.5)	6.5 (0.5)	0.888
Hospitalization per 1000 patient months	6.6 (0.5)	4.7 (0.2)	0.018
Chronic condition-related health expenses (USD $), mean (SE)			
Medical expenses	2990 (206)	1929 (87)	< 0.001
Office	1005 (51)	602 (17)	< 0.001
Outpatient	224 (27)	126 (12)	0.001
ED	91 (13)	82 (4.4)	0.967
Hospitalization	1104 (131)	826 (69)	0.103
Home health	565 (131)	293 (32)	< 0.001
Prescription drugs	1123 (113)	784 (33)	< 0.001
Total expenses	4113 (242)	2714 (103)	< 0.001

*ED* emergency department; *SE* standard error; *USD* United States Dollar

Table 3 displays the all-cause health care utilization patterns and expenses by chronic condition in the subgroup of adults with cancer. Overall, the chronic condition group showed higher total health care utilization than the reference group without chronic conditions, including numbers of office visits, outpatient visits, prescription fills, days provided by home health, and events for ED and hospitalization. The total health care expenses in the chronic condition group were $6388 higher ($13,557 vs. $7169, *p* < 0.001) than the reference group without chronic condition(s).

**Table 3:** All-cause health service utilization patterns and expenses (per-person per-year) by chronic condition in adults with cancer (n = 3657)

	Chronic condition (n = 3040)	No chronic condition (n = 617)	*p*
All-cause health service utilization, mean (SE)			
Number of office visits	14.3 (0.3)	7.5 (0.4)	< 0.001
Number of outpatient visits	1.7 (0.1)	0.9 (0.1)	< 0.001
Number of prescription fills	29.8 (0.8)	6.7 (0.5)	< 0.001
Home health days per month	5.2 (0.8)	0.4 (0.1)	< 0.001
ED visits per 1000 patient months	27 (1.1)	12 (1.3)	< 0.001
Hospitalization per 1000 patient months	23 (1.1)	11 (1.4)	< 0.001
All-cause health expenses (USD $),mean (SE)			
Medical expenses	10,573 (423)	6147 (575)	< 0.001
Office	3499 (110)	2155 (201)	< 0.001
Outpatient	1507 (118)	1038 (151)	< 0.001
ED	332 (26)	186 (34)	< 0.001
Hospitalization	3885 (267)	2198 (467)	< 0.001
Home health	721 (206)	49 (17)	< 0.001
Other	630 (25)	521 (43)	< 0.001
Prescription drugs	2984 (127)	1022 (216)	< 0.001
Total expenses	13,557 (465)	7169 (630)	< 0.001

*ED* emergency department; *SE* standard error; *USD* United States Dollar

Table 4 showed that, after controlling for study sample characteristics, cancer increased chronic condition-related health expenses by 40.3% (*p* < 0.001). In the subgroup of adults with cancer, chronic condition(s) increased total health expenses by 50.7% (*p* < 0.001) after adjustment. The full regression results can be found in Additional files 3 and 4.

**Table 4:** Associations of chronic conditions on total health expenses and effect of cancer on chronic condition-related health expenses

Dependent variable	Independent variable	Estimated log coefficient (SE)	Change in percent (%)^a^	*p*
Chronic condition-related expenses^b^	Cancer			
	No	Reference		
	Yes	0.34 (0.08)	+ 40.3%	< 0.001
Total health expenses^c^	Chronic condition			
	No	Reference		
	Yes	0.41 (0.10)	+ 50.7%	< 0.001

*SE* standard error

^a^The log coefficients of the independent variables were interpreted as the change in percent

^b^The study sample (*n* = 28,108) included eligible adults with chronic conditions. Primary independent variable was cancer (yes/no). Covariates in the regression model for adjustment included respondents’ sociodemographic and health characteristics

^c^The study sample (n = 3657) included a subgroup of adults with cancer. Primary independent variable was chronic condition (yes/no). Covariates in the regression model for adjustment included respondents’ sociodemographic and health characteristics

## Discussion

This study adds to our understanding of the treated prevalence of MCC and associated health services utilization and annual health care expenses among those with cancer compared to the general non-cancer US population. Hypertension and hyperlipidemia were the most prevalent among both those with and without cancer, while arthritis, diabetes, and coronary artery disease, were the third, fourth, and fifth most prevalent among those with cancer, respectively. While depression, diabetes, and arthritis took the third, fourth, and fifth spots for those without cancer, the treated prevalence for all of the ten chronic conditions were significantly greater among those with cancer. The effects of cancer occurrence and treatment might contribute to the high risk of chronic conditions. This suggests that cancer care should address the complex nature of MCC in any treatment plan and that there may be a greater need for coordinated care between specialists and primary care providers to fully address underlying chronic conditions. Preventing recurrent cancer often is one of the primary goals in disease management for cancer patients. However, people living with cancer are at risk for developing comorbid chronic conditions. Helping cancer patients understand and manage comorbid chronic conditions should be considered part of a holistic treatment plan. The differences in patient characteristics (e.g., age, income, education, insurance coverage) between the groups with and without chronic conditions in our study would help health care providers to identify high-risk patients and barriers to chronic condition management.

Our study found the percentage of those with > 4 chronic conditions was about 30% in those with cancer vs. 10% in those with no cancer. In addition, fewer patients with cancer (16%) showed no chronic conditions as compared to those patients without cancer (48%). This added layer of complexity could have an impact on the patient experience with health care providers. MEPS reported that only 54% of adults with MCC felt that physicians spent enough time with them during office visits [20]. Evidence showed that the average duration for outpatient physician office visits for cancer patients was 22.9 min [21]. However, no study has explored visit duration among cancer patients with MCC. More research is needed to examine the effect of disease complexity on visit duration and whether physicians spend enough time addressing comorbidities. Assessing the impact of advanced practice providers in augmenting physician visit time on patient experience or health outcomes is another area for future research. In addition to focusing on cancer management, effective communication between physicians and patients about comorbid chronic conditions is also important in improving patient satisfaction and treatment outcomes. Determining if adequate communication exists between the referring physician and cancer specialist, with corresponding timely follow-up for chronic disease management, may also be an important area to explore.

Several studies used different approaches to define and measure chronic conditions in cancer populations. In prior studies, higher health care costs were significantly associated with chronic conditions, particularly mental health conditions [9, 22]. The OASH list of chronic conditions narrows the chronic condition classification scheme to reflect the HHS strategic framework for MCC, and assists researchers in assessing outcomes associated with chronic conditions by using the HHS data system [18]. The economic burden of cancer and chronic conditions is considerable for both patients and payers. Our study indicated that in the cancer group, health expenses associated with chronic conditions accounted for one-third of total health care expenses and were 40% higher than in the non-cancer group. Additionally, expenses for physician office visits and prescription drug use were major components, accounting more than 50% of the chronic condition-related expenses. These findings suggested the important role access to primary and specialty care plays in managing cancer and MCC. The 2015 US National Health Interview Survey reported that 17% of adults (18–64 years) with MCC delayed or did not obtain needed medical care due to cost, and 20% delayed or did not seek care due to a non-cost reason. These figures were 11.7 and 8.3% higher than those aged 65 years and older, respectively. The out-of-pocket expenses (per-person, per-year) in adults with MCC were 2.2 times higher than in those with one or no chronic conditions [23]. These disparities of out-of-pocket expenses for MCC in race, income, and insurance coverage were identified in a previous study [23]. Cancer patients with MCC represent a complex population. Coordinated care among providers with individualized disease management could help optimize treatment, control health care expenses, and improve quality of life. Our study also indicated that, based on average annual cost per-person, common chronic conditions might not represent costlier conditions. However, the high prevalence of common chronic conditions may result in high total health care expenses for payers in the cancer population.

Several limitations should be considered when interpreting these results. First, this study used MEPS variables for provider-verified diagnosis codes instead of variables for self-reported cancer and chronic conditions. Individuals included in the study sample represent the non-institutionalized population living with cancer and seeking cancer treatment. The prevalence of chronic conditions reflects treated prevalence based on reported health care utilization. Thus, this study should not be used to report disease prevalence and could also underestimate disease prevalence. Second, average health care expenses were estimated based on two-year health care utilization and represented a snapshot of a short-term effect of chronic conditions in adults with cancer. The temporal relationship between chronic conditions and cancer was not clear in the study. Whether the chronic conditions were associated with side effects of cancer treatment, other clinical characteristics, or behavioral factors was not determined. Moreover, the MEPS data did not provide clinical information related to medical conditions to help assess the severity of chronic conditions or cancer stage (e.g., laboratory or diagnostic data). Thus, our analysis was not able to control the unmeasured severity of diseases in the regression models. Further subgroup analysis was not conducted due to the small sample size of each different cancer. Finally, our study only measured direct health care costs associated with chronic conditions. The indirect costs measured by lost productivity, representing the other important aspect in health service research, were not assessed.

## Conclusion

This study provided important implications for health care providers who manage MCC in the cancer population. First, chronic conditions increase patient complexity in those with cancer. Making an optimal holistic treatment plan to improve both health outcomes and quality of life has been a challenge for health care providers. Primary care providers and oncologists play an important role in managing complex patients, and should be aware of the effect of a cancer diagnosis or treatment on pre-existing chronic conditions and potential risk for developing new chronic conditions. Effective communication, care coordination, and increased time with patients during office visits could improve the patient’s experience and outcomes. Second, identifying barriers to health care, including the cost, burden, and complexity of care for cancer patients with MCC would help prioritize disease management and facilitate a patient-centered approach. Education and support systems that address chronic conditions could be an effective means of mitigating the complexity of health issues these patients face [24]. Finally, future research should continue to explore collaborative patient-centered practice models that address MCC in the presence of cancer and ways to mitigate burdensome costs and health care utilization. With an aging US population and the higher prevalence of chronic conditions in adults with cancer, delivering comprehensive and individualized care is not only important to efficiently achieve treatment goals, but also to effectively reduce health care costs.

## Supplementary information

**Additional file 1. **Prevalence of all chronic conditions by cancer (*n*=54,921)

**Additional file 2. **Annual health expenses (per-person) associated with chronic conditions by cancer (*n*=54,921)

**Additional file 3. **Association between of cancer on chronic condition-related health expenses in adults with chronic conditions (*n* = 28,108)

**Additional file 4. **Association between chronic conditions on total health expenses after adjusting for demographics and health characteristics in adults with cancer (*n*=3,657)

## Data Availability

The datasets used and analyzed during the current study are publically available and freely accessible through the Agency for Healthcare Research and Quality (AHRQ) Medical Expenditure Panel Survey (MEPS) homepage in the data files tab on the website (https://meps.ahrq.gov/mepsweb/).

## Abbreviations

AHRQ: Agency for Healthcare Research and Quality
CCS: Clinical Classifications Software
ED: Emergency department
HCUP: Healthcare Cost and Utilization Project
HHS: The US Department of Health and Human Services
ICD-9-CM: International Classification of Disease, Ninth Revision, Clinical Modification
MCC: Multiple chronic conditions
MEPS: Medical Expenditure Panel Survey
OASH: Office of the Assistant Secretary of Health
