# Supplementary material for: Multiple chronic conditions and associated health care expenses in US adults with cancer: a 2010–2015 Medical Expenditure Panel Survey study
Source: BMC Health Serv Res. 2019 Dec 19;19:981. doi: 10.1186/s12913-019-4827-1 (PMC6924021; doi:10.1186/s12913-019-4827-1)
Supplement: Supplementary file 1 — Additional file 1. Prevalence of all chronic conditions by cancer (n=54,921) [file 12913_2019_4827_MOESM1_ESM.docx]

Additional file 1 Prevalence of all chronic conditions by cancer (n=54,921)

| Chronic condition | Prevalence, n (weighted %) | |  |
| --- | --- | --- | --- |
|  | Cancer (n=3,657) | No cancer (n=51,264) | *p* |
| Hypertension | 2,129 (59.7) | 14684 (28.8) | <0.001 |
| Hyperlipidemia | 1,903 (53.6) | 11440 (23.7) | <0.001 |
| Arthritis | 899 (25.6) | 4728 (10.0) | <0.001 |
| Diabetes | 884 (22.2) | 6265 (11.1) | <0.001 |
| Coronary artery disease | 657 (18.2) | 2973 (6.1) | <0.001 |
| Depression | 613 (17.1) | 5319 (12.4) | <0.001 |
| COPD | 545 (15.1) | 3405(7.8) | <0.001 |
| Cardiac arrhythmias | 368 (11.5) | 1583 (3.6) | <0.001 |
| Asthma | 434 (11.1) | 3811 (7.8) | <0.001 |
| Stroke | 236 (6.5) | 1056 (2.1) | <0.001 |
| Osteoporosis | 169 (4.6) | 606 (1.3) | <0.001 |
| Congestive heart failure | 102 (2.7) | 424 (0.9) | <0.001 |
| Dementia | 104 (2.8) | 471 (1.0) | <0.001 |
| Hepatitis | 30 (0.8) | 200 (0.4) | 0.015 |
| Substance abuse | 29 (0.8) | 403 (0.8) | 0.433 |
| Schizophrenia | 28 (0.8) | 253 (0.4) | 0.049 |
| HIV | 21 (0.4) | 122 (0.2) | 0.005 |
| Chronic kidney disease | 8 (0.2) | 45 (0.1) | 0.038 |

COPD: Chronic Obstructive Pulmonary Disease; HIV: human immunodeficiency virus infection; SE: standard error
