# Supplementary material for: Multiple chronic conditions and associated health care expenses in US adults with cancer: a 2010–2015 Medical Expenditure Panel Survey study
Source: BMC Health Serv Res. 2019 Dec 19;19:981. doi: 10.1186/s12913-019-4827-1 (PMC6924021; doi:10.1186/s12913-019-4827-1)
Supplement: Supplementary file 2 — Additional file 2. Annual health expenses (per-person) associated with chronic conditions by cancer (n=54,921) [file 12913_2019_4827_MOESM2_ESM.docx]

Additional file 2 Annual health expenses (per-person) associated with chronic conditions by cancer (n=54,921)

| Chronic condition | Annual health expenses (USD $) per-person,  mean (SE) | |  |
| --- | --- | --- | --- |
|  | Cancer (n=3,657) | No cancer (n=51,264) | *p* |
| Hypertension | 913 (70) | 690 (33) | 0.002 |
| Hyperlipidemia | 636 (58) | 550 (24) | 0.018 |
| Arthritis | 1,052 (107) | 1,216 (90) | 0.203 |
| Diabetes | 1,849 (216) | 1,766 (134) | 0.001 |
| Coronary artery disease | 2,787 (276) | 3,221 (187) | 0.072 |
| Depression | 1,221 (240) | 936 (67) | 0.169 |
| COPD | 1,382 (188) | 1,012 (90) | 0.001 |
| Cardiac arrhythmias | 2,011 (271) | 2,157 (242) | 0.010 |
| Asthma | 894 (138) | 635 (57) | 0.018 |
| Stroke | 2,964 (1,883) | 3,675 (361) | 0.238 |
| Osteoporosis | 839 (169) | 615 (88) | 0.253 |
| Congestive heart failure | 5,541 (1,322) | 5,064 (1,190) | 0.244 |
| Dementia | 3,691 (810) | 3,107 (359) | 0.099 |
| Hepatitis | 4,714 (2,586) | 4,044 (1,273) | 0.035 |
| Substance abuse | 710 (410) | 1,849 (280) | 0.019 |
| Schizophrenia | 7,259 (3,160) | 3,687 (428) | 0.015 |
| HIV | 9,650 (3,409) | 7,338 (1,195) | 0.526 |
| Chronic kidney disease | 3,365 (2,851) | 12,233 (3,741) | 0.035 |

COPD: Chronic Obstructive Pulmonary Disease; HIV: human immunodeficiency virus infection; SE: standard error; USD: United States Dollar
