# Supplementary material for: Multiple chronic conditions and associated health care expenses in US adults with cancer: a 2010–2015 Medical Expenditure Panel Survey study
Source: BMC Health Serv Res. 2019 Dec 19;19:981. doi: 10.1186/s12913-019-4827-1 (PMC6924021; doi:10.1186/s12913-019-4827-1)
Supplement: Supplementary file 3 — Additional file 3. Association between of cancer on chronic condition-related health expenses in adults with chronic conditions (n = 28,108) [file 12913_2019_4827_MOESM3_ESM.docx]

Additional file 3 Association between of cancer on chronic condition-related health expenses in adults with chronic conditions (n = 28,108)

| Variable | Estimated log coefficient (SE) | Change in percent (%)^a^ | *p* |
| --- | --- | --- | --- |
| Cancer |  |  |  |
| No | Reference |  |  |
| Yes | 0.34 (0.08) | +40.5% | <0.001 |
| Age |  |  |  |
| 18-45 | Reference |  |  |
| 46-64 | 0.47 (0.07) | +60.0% | <0.001 |
| 65+ | 0.78 (0.07) | +118% | <0.001 |
| Race |  |  |  |
| White | Reference |  |  |
| Black | 0.12 (0.07) | +12.7% | 0.068 |
| Other | -0.16 (0.05) | -14.8% | 0.003 |
| Sex |  |  |  |
| Male | Reference |  |  |
| Female | -0.24 (0.05) | -21.3% | <0.001 |
| Body mass index |  |  |  |
| Underweight | 0.56 (0.21) | +75.1% | 0.006 |
| Normal | Reference |  |  |
| Overweight | -0.06 (0.07) | -5.8% | 0.395 |
| Obese | 0.07 (0.06) | +7.3% | 0.253 |
| Education |  |  |  |
| High School | Reference |  |  |
| College | 0.07 (0.06) | +7.3% | 0.271 |
| No degree/other | 0.07 (0.06) | +7.3% | 0.232 |
| Family size |  |  |  |
| ≤ 2 | Reference |  |  |
| >2 | -0.12 (0.06) | -11.3% | 0.049 |
| Geographical region |  |  |  |
| Northeast | Reference |  |  |
| Midwest | -0.33 (0.09) | -28.1% | <0.001 |
| South | -0.30 (0.08) | -25.9% | <0.001 |
| West | -0.39 (0.08) | -32.3% | <0.001 |
| Marital status |  |  |  |
| No | Reference |  |  |
| Yes | -0.11 (0.06) | -10.4% | 0.044 |
| Income category^b^ |  |  |  |
| Poor | Reference |  |  |
| Low income | -0.13 (0.09) | -12.2% | 0.152 |
| Middle income | -0.28 (0.07) | -24.4% | <0.001 |
| High income | -0.30 (0.08) | -25.9% | <0.001 |
| Insurance coverage |  |  |  |
| Private | Reference |  |  |
| Public | 0.21 (0.06) | +23.4% | 0.001 |
| Uninsured | -0.41 (0.11) | -33.6% | <0.001 |
| Perceived health status |  |  |  |
| Fair/poor | Reference |  |  |
| Good | -0.77 (0.07) | -53.7% | <0.001 |
| Excellent/Very good | -1.33 (0.07) | -73.6% | <0.001 |

SE: standard error

^a^The log coefficients of the independent variables were interpreted as the change in percent.

^b^Poor defined as income <100% of federal poverty line (FPL); low income defined as 100%-199% of FPL; middle income defined as 200%-399% of FPL; high income defined as ≥ 400% of FPL.
