# Supplementary material for: Multiple chronic conditions and associated health care expenses in US adults with cancer: a 2010–2015 Medical Expenditure Panel Survey study
Source: BMC Health Serv Res. 2019 Dec 19;19:981. doi: 10.1186/s12913-019-4827-1 (PMC6924021; doi:10.1186/s12913-019-4827-1)
Supplement: Supplementary file 4 — Additional file 4. Association between chronic conditions on total health expenses after adjusting for demographics and health characteristics in adults with cancer (n=3,657) [file 12913_2019_4827_MOESM4_ESM.docx]

Additional file 4 Association between chronic conditions on total health expenses after adjusting for demographics and health characteristics in adults with cancer (n=3,657)

| Variable | Estimated log coefficient (SE) | Change in percent^a^ | *p* |
| --- | --- | --- | --- |
| Chronic condition |  |  |  |
| No | Reference |  |  |
| Yes | 0.41 (0.10) | +50.7% | <0.001 |
| Age |  |  |  |
| 18-45 | Reference |  |  |
| 46-64 | 0.30 (0.13) | +35.0% | 0.025 |
| 65+ | 0.08 (0.14) | +8.3% | 0.574 |
| Race |  |  |  |
| White | Reference |  |  |
| Black | 0.23 (0.09) | +25.9% | 0.013 |
| Other | 0.06 (0.07) | +6.2% | 0.422 |
| Sex |  |  |  |
| Male | Reference |  |  |
| Female | -0.15 (0.07) | -13.9% | 0.031 |
| Body mass index |  |  |  |
| Underweight | 0.16 (0.15) | +17.4% | 0.280 |
| Normal | Reference |  |  |
| Overweight | 0.04 (0.08) | +4.1% | 0.641 |
| Obese | 0.15 (0.08) | +16.2% | 0.051 |
| Education |  |  |  |
| High School | Reference |  |  |
| College | -0.07 (0.10) | -6.8% | 0.490 |
| No degree/other | -0.03 (0.08) | -3.0% | 0.688 |
| Family size |  |  |  |
| ≤ 2 | Reference |  |  |
| >2 | -0.18 (0.08) | -16.5% | 0.023 |
| Geographical region |  |  |  |
| Northeast | Reference |  |  |
| Midwest | 0.02 (0.09) | +2.0% | 0.845 |
| South | -0.15 (0.08) | -13.9% | 0.074 |
| West | -0.07 (0.09) | -6.8% | 0.404 |
| Marital status |  |  |  |
| No | Reference |  |  |
| Yes | -0.04 (0.08) | -3.9% | 0.620 |
| Income category^b^ |  |  |  |
| Poor | Reference |  |  |
| Low income | 0.24 (0.12) | +27.1% | 0.046 |
| Middle income | 0.12 (0.09) | +12.7% | 0.167 |
| High income | 0.11 (0.08) | +11.6% | 0.301 |
| Insurance coverage |  |  |  |
| Private | Reference |  |  |
| Public | -0.01 (0.07) | -0.01% | 0.960 |
| Uninsured | -0.87 (0.16) | -58.1% | <0.001 |
| Perceived health status |  |  |  |
| Fair/poor | Reference |  |  |
| Good | -0.38 (0.07) | -31.6% | <0.001 |
| Excellent/Very good | -0.66 (0.08) | -48.3% | <0.001 |

SE: standard error

^a^The log coefficients of the independent variables were interpreted as the change in percent.

^b^Poor defined as income <100% of federal poverty line (FPL); low income defined as 100%-199% of FPL; middle income defined as 200%-399% of FPL; high income defined as ≥ 400% of FPL.
